# Genetic nurse counsellors can be an acceptable and cost-effective alternative to clinical geneticists for breast cancer risk genetic counselling. Evidence from two parallel randomised controlled equivalence trials

**DOI:** 10.1038/sj.bjc.6603248

**Published:** 2006-07-11

**Authors:** N Torrance, J Mollison, S Wordsworth, J Gray, Z Miedzybrodzka, N Haites, A Grant, M Campbell, M S Watson, A Clarke, B Wilson

**Affiliations:** 1 Department of Public Health, Medical School, University of Aberdeen, Foresterhill, Aberdeen AB25 2ZD, UK; 2 Health Economics Research Centre, University of Oxford, Oxford OX3 7LF, UK; 3 Institute of Medical Genetics, University Hospital of Wales, Cardiff CF14 4XN, UK; 4 Department of Medicine & Therapeutics, University of Aberdeen, Aberdeen AB25 2ZD, UK; 5 Health Services Research Unit, University of Aberdeen, Aberdeen AB25 2ZD, UK; 6 Department of Epidemiology & Community Medicine, University of Ottawa, 451 Smyth Road, Ottawa, Ontario, Canada K1H 8M5

**Keywords:** genetic counselling, familial cancer, breast cancer, randomised control trial, equivalence, anxiety

## Abstract

This study compared genetic nurse counsellors with standard services for breast cancer genetic risk counselling services in two regional genetics centres, in Grampian region, North East Scotland and in Cardiff, Wales. Women referred for genetic counselling were randomised to an initial genetic counselling appointment with either a genetic nurse counsellor (intervention) or a clinical geneticist (current service, control). Participants completed postal questionnaires before, immediately after the counselling episode and 6 months later to assess anxiety, general health status, perceived risk and satisfaction. A parallel economic evaluation explored factors influencing cost-effectiveness. The two concurrent randomised controlled equivalence trials were conducted and analysed separately. In the Grampian trial, 289 patients (193 intervention, 96 control) and in the Wales trial 297 patients (197 intervention and 100 control) returned a baseline questionnaire and attended their appointment. Analysis suggested at least likely equivalence in anxiety (the primary outcome) between the two arms of the trials. The cost per counselling episode was £11.54 less for nurse-based care in the Grampian trial and £12.50 more for nurse-based care in Cardiff. The costs were sensitive to the grade of doctor (notionally) replaced and the extent of consultant supervision required by the nurse. In conclusion, care based on genetic nurse counsellors was not significantly different from conventional cancer genetic services in both trial locations.

Familial cancer is an area in which genetic knowledge has progressed rapidly over the past 10 years, and where patient demand for genetics services for information, counselling and mutation testing has increased dramatically. Even with the introduction of referral guidelines, regional genetics clinics are facing the challenge of increasing demand within fixed resources (Kinmonth *et al*, 1998), and these difficulties may be exacerbated as new mutations are identified and further demands are made on genetics clinics.

Breast cancer is the most common cancer affecting UK women, resulting in a lifetime risk of one in nine of developing the disease (Office for National Statistics, 2005). The discovery of breast cancer susceptibility mutations has attracted widespread publicity and women with a family history of breast cancer dominate referrals throughout the UK for any cancer genetic risk counselling (Wonderling *et al*, 2001; Hopwood *et al*, 2004). Mutations in the known inherited susceptibility genes, *BRCA1* and *BRCA2*, give rise to increased lifetime risks of developing the disease, often at an earlier age than sporadic disease (Claus *et al*, 1994). However, these mutations are implicated in around only 5–10% of breast cancer cases, with the result that genetic testing is not appropriate for the majority of women with a family history of breast cancer. Genetic counselling services aim to identify individuals who have a significantly increased genetic risk of cancer and counsel them about appropriate risk management to reduce morbidity and mortality (Fry *et al*, 2003). Access to surveillance, testing and other interventions is usually dependent on the outcome of the initial genetic counselling and risk assessment.

Recent research in the area of breast cancer genetic risk counselling has generally concentrated on women's psychological status and risk perception. Evidence from systematic reviews suggests that genetic counselling does not appear to have any adverse effect on psychological outcomes (Meiser and Halliday, 2002; Butow *et al*, 2003; Braithwaite *et al*, 2004), and may convey some short-term benefits in decreasing general anxiety and in improving the accuracy of women's perceptions of their personal risk. Historically, clinical genetic risk assessments in the UK have been conducted largely by medical doctors, with a variable amount of support for genetic counselling provided by genetic nurses and counsellors, social workers and psychologists as well. Many specialist centres have recently begun to expand their genetic risk counselling capacity by recruiting genetic nurses or associates (Skirton *et al*, 1998). This trend is likely to continue. However, there is a lack of formal evaluation of the effectiveness of this strategy and the lack of well designed and conducted randomised controlled trials of genetic service models has been noted in a number of recent publications (Butow *et al*, 2003; Braithwaite *et al*, 2004; Hopwood *et al*, 2004; NICE, 2004).

We therefore conducted a pragmatic evaluation comparing genetic nurse counsellors as an alternative to a physician-based service (standard care) in assessing patients newly referred for genetic counselling for risk of breast cancer. We focused on patient-centred outcomes, and acceptability to referring general practitioners, and conducted a concurrent economic evaluation. In order to increase the generalisability of our findings, we conducted the evaluation as two randomised controlled equivalence trials with differing patient population and service characteristics.

## MATERIALS AND METHODS

### Participants and procedures

Two concurrent randomised controlled trials were conducted to assess the equivalence of care provided by a genetic nurse counsellor with clinical geneticist-based (standard) care. The study locations were the Grampian University Hospitals NHS Trust Clinical Genetics Service (in Aberdeen) and the Wales Genetics Service (in Cardiff). The study was designed with a pragmatic approach; that is, the goal was to gain an insight into the overall effects of substituting a nurse counsellor-based approach for a physician-based approach, while accepting that contextual factors inevitably vary between health care settings. Thus, there were differences in regular (doctor-based) care in the two trial locations, but both represented an acceptable ‘standard of care’ for breast cancer genetic risk assessment and counselling within the NHS. Similarly, the intervention arms reflected nurse counsellor-based care developed and managed according to local protocols and supervisory arrangements. As a clinical geneticist was formally responsible in each location for patient care, whether it was delivered by a doctor or a nurse counsellor, we were not concerned with assessing performance of the nurse counsellors in relation to technical procedures such as pedigree construction or calculation of risk estimates. Rather, we concentrated on other outcomes of interest, including provider and patient satisfaction. By implementing trial procedures rigorously, collecting identical data sets, but analysing the two trials separately, we were able to promote internal validity within each trial but also comment on external validity (i.e. generalisability) (Pocock, 1983).

Patients were eligible if they were newly referred because of concern about family history of breast cancer, were aged 18 years or over, and literate in English. Patients previously affected by breast cancer were included. Patients were excluded if they had previously attended the genetics clinic or were known to be a member of a family in which a *BRCA1* or *BRCA2* mutation had been previously identified.

Eligible patients were identified from referral letters and consent to contact was obtained from each patient's GP. Before their clinic appointment, patients were sent a letter inviting their participation, an information sheet explaining the study and a consent form. Those who consented were randomised to the intervention (genetic nurse counsellor-based care) or control group (clinical geneticist-based care). The random allocation schedule sequence for each trial was computer-generated and concealed within a Microsoft Access database. To avoid possible contamination, members of the same family were allocated to the same trial group. Eligible patients were randomised 2 : 1 to intervention: control groups. This uneven allocation was chosen to give greater experience of the novel intervention and was taken account of in the sample size calculation. Postal questionnaires were administered to participants at baseline (before the clinic appointment), immediately following the counselling episode (FU1), and 6 months later (FU2).

### Standard care in two trial locations (clinical geneticist)

As indicated above, the control arms reflected current standard of care in each location, and no attempt was made to alter this. The process for patients referred for genetic assessment was similar in both centres: family history taking, pedigree construction, confirmation of cancer diagnoses in affected relatives, risk assessment, genetic counselling with advice on preventive strategies and, if appropriate screening, prophylactic surgery, and/or genetic testing. Initially, the clinical geneticist took the family history, constructed the pedigree, confirmed cancer diagnoses, etc in both centres. In Grampian, clerical and nursing support was available to enable the family tree to be constructed in advance of the clinic appointment. In Wales the clinical geneticist did all of the preclinic preparation. In both settings, clinic appointments involved a clinical geneticist and lasted approximately 45 min.

### Intervention (genetic nurse counsellors)

In both trials, the nurse counsellor made the initial contact with the patient. Each followed the format of the standard care described above for their own centre, with a nurse counsellor substituting for clinical geneticist input. Each clinic was therefore free to make its own organisational arrangements for the nurse-led counselling arm, so long as there were adequate minimum supervision arrangements, a clinical geneticist was professionally and managerially accountable for the patient care delivered by a nurse counsellor, and the same risk assessment algorithms were followed *within* each clinic (by both nurse counsellors and clinical geneticists). The focus of the study was the ‘counselling episode’: the time from appointment scheduling until the patient was either discharged or further management arrangements were made. This provided the ‘boundary’ to the intervention – all activities from this point on were part of the ‘usual’ cancer genetics service. Participants who were considered likely candidates for genetic testing (i.e. high risk) were referred for further follow-up with a consultant geneticist. In both trials, a consultant geneticist actively supervised the nurse counsellors, meeting with them on a weekly basis. Training was delivered according to the needs of the nurse counsellors when they were appointed.

### Data collection

#### Sociodemographic data

Sociodemographic data were collected from the women in the baseline questionnaire, and included age, marital status, number of children, ethnicity and educational level.

#### Psychological outcomes

The primary outcome was patient anxiety, measured using the six-item short-form of the state scale of the Spielberger State-Trait Anxiety Inventory (STAI; Marteau and Bekker, 1992). The short version produces result comparable with the full state scale and has been used in other genetic counselling research (Miedzybrodzka *et al*, 1995; Bish *et al*, 2002). We also included the Hospital Anxiety and Depression Scale (HADS; Zigmond and Snaith, 1983), which gives separate measures of anxiety and depression assessed over the past week (range of scores from 0 to 21, seven items for anxiety and seven for depression).

#### Health-related quality of life

The Short Form 36 (SF-36) health survey instrument was used to measure perception of general health status (Ware *et al*, 1994). It measures patients' rating of their own health status in eight areas or domains: physical functioning, role physical, role emotional, social functioning, mental health, vitality, bodily pain and general health perception. For each dimension item scores range from 0 (worst possible health state) to 100 (best possible health state).

#### Perceived risk of breast cancer

We assessed participants' perceived risk of breast cancer at all three time points with an item used in previous research (Lerman *et al*, 1993; Lloyd *et al*, 1996; Watson *et al*, 1999; Brain *et al*, 2000). Women were asked to assess their own risk of developing breast cancer relative to a notional ‘average’ woman, on a five-point scale (‘much lower/lower/about the same/higher/much higher’). Women previously affected by breast cancer were asked to skip this section of the questionnaire to avoid potential confusion.

#### Knowledge of breast cancer risk factors

We assessed participants' understanding of risk factors for breast cancer at all three time points. Women were asked how strongly they agreed with three statements on specific causes of breast cancer (stress, having a first-degree relative with breast cancer and minor injury). We used a five-point scale (strongly disagree/disagree/not sure/agree/strongly agree). For each of these three potential risk factors, agreement would indicate a response inconsistent with the education content offered during the genetic counselling appointment.

#### Patient satisfaction and acceptability to referring GPs

Patient satisfaction was measured using a modified version of the Satisfaction with Genetic Counselling Questionnaire developed by Shiloh *et al* (1990) and was incorporated into both follow-up questionnaires. The scale assesses three dimensions of patient satisfaction: (1) instrumental (satisfaction with the doctor/nurse's competence), (2) affective (satisfaction with the doctor/nurse's personal qualities) and (3) procedural (satisfaction with administrative procedures, such as waiting time and staff conduct). The acceptability of the genetic nurse counsellors to referring GPs was assessed towards the end of the study. A short questionnaire was used to ascertain whether the referring GPs had noticed any difference (including deterioration) in the service provided by the genetics department for their patients, if they would be happy for their patients to be seen by a genetic nurse counsellor in the future and an overall rating of their satisfaction with the genetics services.

### Statistical analysis

Descriptive statistics were used to describe the study participants. As this was an equivalence trial, a confidence interval (CI) approach was used (Jones *et al*, 1996). The difference in outcomes was calculated, adjusting for differences in baseline scores using multiple linear regression. For each outcome, the 95% CIs around the difference were calculated. An outcome was considered ‘equivalent’ when the 95% CI for the difference between the intervention and control group fell completely within a predetermined equivalence limit (Jones *et al*, 1996). Where the 95% CI for the observed difference fell completely outside the equivalence limit, we considered the outcomes would be ‘nonequivalent’. Where the 95% CI for the observed difference overlapped the equivalence limit, the result would be uncertain. For the primary outcome, the STAI score, we defined an *a priori* strict limit of ‘equivalence’ of ±4 units (used in the sample size calculation below), and a ‘likely equivalence’ limit of ±10 units (which the trial steering group considered more reflective of actual clinical practice). The strict limit was defined before the study started, and the likely equivalence limit was determined after the study began, but before data were released for analysis. For anxiety as measured by HADS, and the role-emotional and mental health domains of the SF36, we defined the equivalence limit as one-third of a standard deviation of the baseline scores.

We analysed data using the ‘intention to treat’ principle, in order to minimise the effect of selection bias and tested the robustness of the findings by comparing the findings to a ‘treatment-received’ analysis. Analysis was performed using SPSS v11.0.

#### Sample size

The primary outcome was anxiety as assessed by STAI (short form), with an equivalence limit of ±4 units. We required 214 participants in the intervention group and 107 in the control group to allow detection of equivalence (two sided), with 80% power at the 5% significance level. This assumed a standard deviation of 12 units for STAI (Marteau *et al*, 1990), and incorporated the 2 : 1 allocation ratio.

### Economic evaluation

A cost analysis was conducted alongside the trials, adopting a societal perspective to include health service and patient costs. The ‘cost per patient counselling episode’ for each woman in both trials was calculated. Data on health service resource use (staff, consumables, rooms and equipment) were collected and local unit costs applied where available, otherwise national rates were used. Staff time included outpatient appointments, preparation, meetings and time in the nurse counsellor arm for consultant geneticists discussing patient cases with the nurses. The mid-point of salary scales was used, adding employers' on-costs at 13%. An equivalent annual cost was calculated for equipment items such as computers, over relevant life-spans for the items, using a 6% discount rate. A total cost per counselling episode for each woman was calculated by multiplying the unit cost per single appointment by the number of appointments in each trial arm. Sensitivity analysis tested the robustness of the findings and data on patient time and travel costs were also collected. All costs are reported in Sterling (£) for the price year 2006.

Approval for the study was obtained from the Joint Ethics Committee of Aberdeen University and Grampian Health Board, and the research ethics committees of Bro Taf and Iechyd Morgannwg health authorities.

## RESULTS

### Participants

#### Grampian

**Figure 1:**
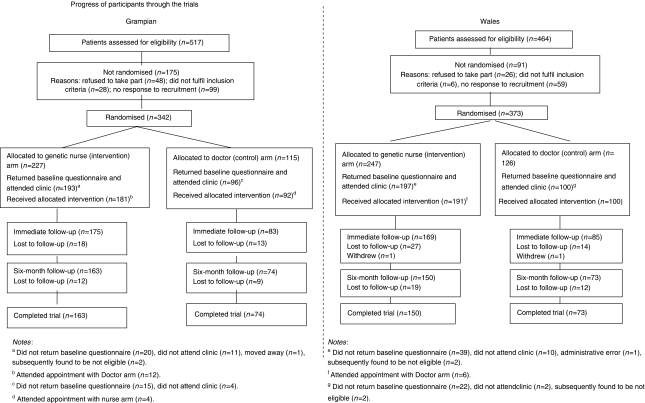
Progress of participants through the trials.

In total, 517 referred patients were considered for recruitment (Figure 1). Of the 342 (66%) patients who consented and were randomised (227 intervention, 115 control), 289 (84%) returned a baseline questionnaire and attended clinic (193 (85%) intervention, 96 (83%) control). In total, 17 did not receive the allocated management because of administrative errors (6), joint family appointments (4) or decision by the head of service (7).

#### Wales

In total, 464 patients were considered for recruitment (Figure 1). Of the 373 (80%) patients who consented and were randomised (247 intervention, 126 control), 297 (80%) returned a baseline questionnaire and attended clinic (197 (80%) intervention, 100 (79%) control). Six women did not receive the allocated management because of administrative errors (2) and joint family appointment (4).

Baseline data are presented for women who returned a baseline questionnaire and attended a clinic appointment (Table 1). The randomised groups were generally similar in terms of demographic characteristics. Within each trial, similar proportions of participants allocated to each arm perceived themselves at elevated risk, although Wales participants were generally more likely to view themselves as at elevated risk than Grampian participants. The actual estimated lifetime risk of breast cancer for participants only became available after each initial individual counselling episode was complete. In the Grampian trial, participants in the intervention arm were more likely than those in the control arm to be assessed as being at elevated risk, whereas the converse was observed for the Wales trial. The analysis by treatment received produced only minor differences in results compared with the analysis by intention-to-treat, therefore the data are not reported in this paper.

### Anxiety and general health status

Table 2 summarises the primary outcomes for the two trials. There were small but consistent baseline differences between the Grampian and Wales study populations, but generally scores were comparable between intervention and control arms within each trial. With respect to STAI, all adjusted point estimates for the differences between intervention and control groups met the definition of, at least, ‘likely equivalence’.

For anxiety and depression as measured by HADS, *a priori* equivalence limits of one-third of the baseline standard deviation were calculated from the data as ±1.4 (Grampian) and ±1.5 (Wales) for anxiety and ±1.2 (both trials) for depression. These are close to the smallest possible difference in score for an individual, which is ±1 point. The results of all analyses were consistent with ‘equivalence’. Also, shown are the observed differences in the SF36 role-emotional and mental health domains. The equivalence limits were calculated as ±11.4 and ±13.1 for the role-emotional score, and ±6.0 and ±6.3 for the mental health scale, for Grampian and Wales, respectively. The data suggested ‘equivalence’ in these outcomes, in both trials, at both follow-up points, with the exception of the mental health score at the second follow-up point in the Grampian trial, which indicated that ‘uncertain equivalence’.

Table 3 summarises the SF36 scores for the other health status outcomes. Scores were generally high, the lowest being observed for the vitality domain in both trials. On average, higher scores were observed in all domains in the Grampian trial compared with the Wales trial.

### Patient knowledge

Table 4 summarises the proportions of participants who agreed or strongly agreed with each statement on a possible cause of breast cancer. The results are generally similar between the two trial locations, and between intervention and control arms within each trial. Misunderstanding was greatest for the effect of having a first-degree relative with breast cancer. No consistent, or statistically significant improvements in knowledge were observed for any of the three notional risk factors.

### Patient satisfaction

**Figure 2:**
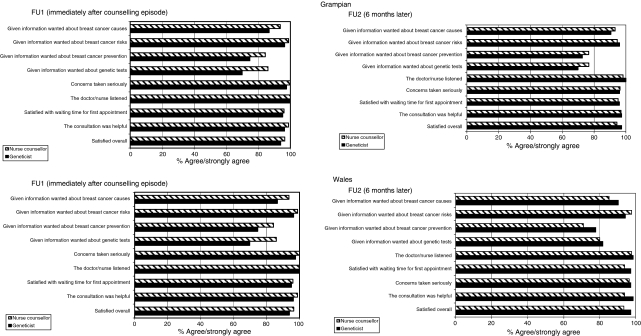
Patient satisfaction with genetic counselling.

High levels of patient satisfaction were observed in both trials. Figure 2 shows the views of patients on the specific aspects of satisfaction with services immediately following the genetic counselling episode and at the 6 month follow-up point.

### Acceptability to referring GPs

In all, 74 and 87 GPs in Grampian and Wales, respectively, referred at least one patient who was randomised to the nurse counsellor and who attended the genetic clinic. In all, 68 GPs in Grampian (response rate 92%) and 75 GPs in Wales (response rate 86%) responded to the acceptability survey. Grampian respondents, 60 (88%) and 52 (69%) Wales respondents could not differentiate whether their patient had been seen by a nurse counsellor or a clinical geneticist. Almost all respondents (Grampian 100%, Wales 98.7% (*n*=74)) reported that they would be happy for future referred patients to be seen at the clinic by the genetic nurse counsellor. Overall satisfaction with the medical genetics service was high with 91% (62 out of 68) GPs in Grampian and 89% (67out of 75). In Wales reporting that they were ‘very satisfied/satisfied’ with the service provided by the respective medical genetics services.

#### Economic evaluation

The unit costs per counselling appointment were similar for the clinical geneticist arms in both trials. Cost differences between intervention and control arms across the two locations were largely driven by staff costs. For Grampian, the marginal (additional) cost per single counselling appointment was £17.98 higher for the control compared with the intervention arm; in Wales, the marginal cost per counselling appointment was £12.50 higher for the intervention than the control arm.

Table 5 presents the health service costs per counselling episode. For Grampian women randomised to the nurse counsellor, the ‘counselling episode’ ranged from 1 to 4 appointments. Most women received one (149 out of 193, 77%) or two (38, 20%) appointments. In the control arm, 81 out of 96 (84%) participants received one, and 13 (13.5%) participants received two, appointments. The mean cost per patient for the counselling episode was £136.55 for the nurse counsellor arm and £148.30 for the clinical geneticist arm, a difference of £11.54 (95% CI, £−25.43, 1.94). In Wales all participants received one appointment only, hence the cost per episode and appointment were the same.

Overall, the cost per counselling episode for the nurse counsellor was £3.55 lower in Grampian compared with Wales, and for the geneticist arm was £20.70 higher in Grampian than in Wales. Health service unit costs in both centres appeared sensitive to grades of staff employed (nurses and doctors), level of supervision of nurse specialists, and length of counselling appointments, but not to choice of discount rate or lifespan of equipment items.

## DISCUSSION

This study suggests that, for the initial episode of genetic counselling for risk of breast cancer, nurse counsellors can provide care that appears to be equivalent to that provided by clinical geneticists, in terms of patients' psychosocial outcomes and satisfaction. The similar findings in the two separate trials support the generalisability of the findings.

Response rates to the baseline and follow-up surveys were high and we were able to assess the stability of the outcomes over a 6-month period following the counselling episode. A high proportion of eligible patients were recruited into both trials, suggesting that our study populations were representative of the target populations. The small but distinct baseline differences between the two sets of trial participants probably reflect real differences in the patient populations from which they were drawn. Participants in Wales had slightly higher anxiety levels overall than the Grampian participants, but these were balanced between intervention and control arms within each setting. After genetic counselling, small reductions in anxiety levels were seen in all groups. Even though the participants' baseline mean anxiety scores were slightly higher than the norm for adult women (score of 35), they were comparable to anxiety levels found in other studies that have used the STAI (Cull *et al*, 1998; Cull *et al*, 1999; Julian-Reynier *et al*, 1999; Brain *et al*, 2000). The between-trial differences support the decision to conduct two parallel trials rather than multicentre trial, where the data would have been pooled (Bowling, 1997). Comparative data for SF-36 scores for these two general geographical populations (Garratt *et al*, 1993; Lyons *et al*, 1995) also suggest that our trial participants were slightly more anxious than the underlying populations, but the between-trial differences were similar to the background between-population differences. It is also possible that these apparent differences reflect the different healthcare referral processes in operation in the two locations. For example, in Wales, women who were considered to be at increased familial risk at the breast screening clinic (Breast Test Wales) were first referred to a surgeon who, after review, made the referral for genetic counselling. In Grampian, many women were referred more directly by GPs or other providers to the genetics clinic. It is possible that these differences in referral pathways contributed to different anxiety levels in women by the time they received a genetics clinic appointment, and also influenced their risk perceptions. Despite these apparent population differences, the effectiveness data from the two trials were very similar.

Few of the participants in these trials would have been eligible for genetic testing, and this was not the focus of the study (which was the period in which initial assessments were made). In both locations, women who were assessed as suitable for genetic testing would have been referred to a consultant geneticist for follow-up care. Our study end point was the point at which a woman learnt of her risk status, and further follow-up or management arrangements were made. We do not have access to data on how many of the participants actually went ahead with genetic testing, or their actual test results.

The primary outcome measure was anxiety, the reduction of which is regarded as a key counselling objective (Shaw *et al*, 1999; Brain *et al*, 2000; Meiser and Halliday, 2002) and a number of evaluations of genetic counselling for familial cancer have identified the pre- and postcounselling assessment of generalised anxiety as a main outcome measure (Cull *et al*, 1998, 1999; Julian-Reynier *et al*, 1999; Brain *et al*, 2000; Kent *et al*, 2000; Bish *et al*, 2002; Bowen *et al*, 2004). Evidence from systematic reviews suggests that, overall, genetic counselling has the effect of significantly reducing patients' anxiety levels, at least in the short-term (Meiser and Halliday, 2002; Butow *et al*, 2003; Braithwaite *et al*, 2004). The *a priori* equivalence limits for the primary outcome (STAI) were set at a very strict level, and in reality they probably represent a smaller difference than would normally be considered clinically significant between two clinicians considered equally competent; however, results showed that some outcomes were considered ‘equivalent’ even at this strict level.

Three common mistaken beliefs on the causes of breast cancer were not influenced by counselling; the most persistent and erroneously held belief was in the influence of one close relative with breast cancer on a person's own risk. This may have reflected a general misconception, or reflect the participants' own personal risk perceptions. The knowledge we assessed was not specific to cancer genetics, and our findings are consistent with genetic counselling delivered by either doctors or nurses being equally effective (or ineffective) in educating patients about breast cancer risk. Braithwaite *et al* (2004) suggest that genetic counselling can be effective in improving knowledge related to breast cancer genetics, compared with no counselling or counselling delivered by nongenetics specialists. However, the observed level of misunderstanding seen in this study suggests that more effective population interventions are required to improve general knowledge of breast cancer risks.

Patient satisfaction with information provided, staff attitudes and the overall clinic procedures were high overall in both trials irrespective of randomised group. Similar levels of patient satisfaction have been reported in other trials of genetic counselling services for risk of breast cancer (Brain *et al*, 2000; Hopwood *et al*, 2004; Holloway *et al*, 2004). In addition, the acceptability of the genetic nurses counsellors was high among referring GPs in both trial settings. However, it is possible that the difference in service model (i.e. appointment of the nurse counsellors) may be imperceptible at primary care level as individual GPs referred only one or two patients during the trial.

The lack of well-designed and rigorously conducted randomised controlled trials, with reporting to CONSORT standards, in the field of service delivery for genetic counselling for familial cancer has been observed in a number of recent publications (Butow *et al*, 2003; Braithwaite *et al*, 2004; Hopwood *et al*, 2004; NICE, 2004). Alternative models for how nurse counsellors might provide a risk assessment service include the concept of working in liaison or outreach settings (Emery *et al*, 1999; Fry *et al*, 1999). Two randomised controlled trials have reported on different models of service delivery for genetic counselling for risk of breast cancer (Brain *et al*, 2000; Fry *et al*, 2003). In the trial by Brain *et al* (2000), the addition of specialist genetic assessment to the standard surgical consultation had no effect on patients' psychological outcomes, risk perception or satisfaction, although knowledge of cancer genetics showed greater improvement. The other RCT found community-based genetic nurse specialists to be generally comparable to the standard service (consultant geneticist), in terms of psychosocial outcomes and patient satisfaction, with the additional benefit of lower staff and patient costs (Fry *et al*, 2003; Holloway *et al*, 2004).

There is now an emerging body of research evidence on the relative effectiveness of nurses in specialist roles compared with doctors in both primary care (Horrocks *et al*, 2002), and secondary care settings (Kinley *et al*, 2001; Sharples *et al*, 2002), with most studies finding that nurses working to guidelines appear to provide care that is equal to that provided by doctors, with comparable health outcomes. Previous randomised trials in secondary care have found nurse practitioners to be either cost neutral (Kinley *et al*, 2001) or more expensive than doctors either because of salary costs (Sakr *et al*, 2003) or greater resource use (Sharples *et al*, 2002). In this study, we found that the relative costs of the nurse counsellors, compared with the doctor-led service, depended on the grade of medical staff whose time was being replaced by the nurse counsellor, and the extent of supervision required – of both nurse counsellors and less experienced medical staff. Surprisingly, the sensitivity analysis did not suggest that nurse counsellors of lower grades would be less costly than the nurses employed here, on the basis that the cost saving in lower salaries would be offset by the greater need for consultant supervision for nurses at lower grades.

The results of this study add to the emerging body of evidence supporting the effectiveness, and possible cost-effectiveness, of nurse counsellors working under the supervision of consultant geneticists, and should be taken into account by decision-makers planning and evaluating genetics health services.

## Figures and Tables

**Table 1:** Characteristics of study participants at baseline

	**Grampian**		**Wales**	
	**Nurse counsellor**	**Geneticist**	**Nurse counsellor**	**Geneticist**
No. of patients	193	96	197	100
Age (years): mean (s.d.)	40.7 (10.3)	41.4 (9.4)	39.8 (10.2)	39.0 (9.3)
Married/cohabiting: *n* (%)	151 (80.3)	74 (77.9)	161 (81.7)	84 (84.8)
With children: *n* (%)	157 (83.5)	73 (76.0)	157 (79.7)	73 (73.0)
Post-secondary education: *n* (%)	76 (39.4)	43 (44.8)	73 (37.1)	42 (42.0)
				
*Referral source: n* (%)				
General practitioner	133 (68.9)	63 (65.6)	117 (59.4)	56 (56.0)
Breast surgeon	35 (18.1)	19 (19.8)	73 (37.1)	42 (42.0)
Breast screening clinic	17 (8.8)	10 (10.4)	—	—
Other	8 (4.1)	4 (4.2)	7 (3.5)	2 (2.0)
				
*n* (%) perceiving themselves to be at elevated risk at baseline	130/181^a^ (72)	65/91^a^ (71)	119/ 179^b^ (83)	76/90^b^ (84)
*n* (%) assessed at clinic at elevated risk	173/192^c^ (90)	74/95^c^ (78)	145/197^d^ (74)	84/100^d^ (84)

For *perceived risk*, participants previously affected by breast cancer are excluded:

a Seven nurse counsellor, one geneticist.

b 10 nurse counsellor, three geneticist.

For *assessed risk*, participants previously affected by breast cancer are included:

c Five nurse counsellor, one geneticist.

d 10 nurse counsellor, three geneticist.

**Table 2:** Psychological outcomes, mean (s.d.)

	**Grampian**				**Wales**			
**Outcomes**	**Nurse counsellor**	**Geneticist**	**Equivalence limit^a^**	**Difference^b^ (95% CI)**	**Nurse counsellor**	**Geneticist**	**Equivalence limit**	**Difference^b^ (95% CI)**
*STAI* c								
Baseline	37.3 (13.6)	36.5 (12.8)			40.9 (15.1)	40.0 (14.5)		
Immediately after episode	36.4 (14.0)	34.4 (14.0)	±4.0	0.8 (−2.1 to 3.7)	38.1 (14.9)	38.9 (15.6)	±4.0	−1.5 (−4.5 to 1.5)
Six months after episode	36.0 (13.5)	32.1 (11.7)		2.9 (−0.2 to 5.9)	38.9 (14.9)	38.1 (14.1)		0.6 (−2.9 to 4.1)
								
*HADS*								
Anxiety^c^								
Baseline	6.7 (4.3)	6.4 (4.5)			8.1 (4.7)	7.4 (4.2)		
Immediately after episode	6.3 (4.3)	5.5 (3.9)	±1.4	0.5 (−0.4 to 1.3)	7.0 (4.9)	7.1 (4.8)	±1.5	−0.4 (−1.3 to 0.5)
Six months after episode	6.2 (4.4)	5.5 (3.7)		0.1 (−0.7 to1.0)	7.4 (4.7)	6.4 (4.1)		0.5 (−0.6 to 1.5)
								
*HADS*								
Depression^d^								
Baseline	3.9 (3.7)	3.4 (3.4)			4.5 (3.7)	4.2 (3.8)		
Immediately after episode	3.5 (3.6)	2.9 (2.8)	±1.2	0.3 (−0.4 to 1.0)	4.0 (3.8)	4.0 (3.8)	±1.2	−0.2 (−1.0 to 0.5)
Six months after episode	3.4 (3.6)	2.8 (2.9)		0.3 (−0.5 to 1.0)	4.5 (4.1)	3.9 (3.8)		0.6 (−0.4 to 1.5)
								
*SF36*								
Role emotional^e^								
Baseline	80.5 (34.6)	82.6 (33.4)			74.4 (38.7)	71.0 (40.6)		
Immediately after episode	81.6 (35.2)	82.5 (33.2)	±11.4	1.9 (−6.3 to 10.1)	74.8 (39.5)	71.5 (40.0)	±13.1	2.9 (−6.9 to 12.7)
Six months after episode	80.3 (35.9)	86.0 (30.7)		−2.5 (−11 to 5.9)	74.9 (38.7)	73.1 (42.2)		0.5 (−9.4 to 10.5)
								
*SF36*								
Mental health^e^								
Baseline	71.0 (18.2)	73.6 (17.7)			67.3 (18.8)	68.4 (19.3)		
Immediately after episode	72.2 (18.6)	74.4 (17.7)	±6.0	0.6 (−2.9 to 4.1)	68.8 (20.5)	68.0 (21.3)	±6.3	1.3 (−2.7 to 5.2)
Six months after episode	72.3 (18.4)	77.4 (14.9)		−2.7 (−6.5 to 1.2)	67.1 (21.1)	67.4 (21.1)		0.3 (−4.2 to 4.8)

a Set at ±4.0 for STAI, 1/3 of baseline s.d.s otherwise.

b Adjusted for baseline.

c Higher score indicates greater level of anxiety.

d Higher score indicates greater level of depression.

e Higher score indicates better health state.

**Table 3:** Health related quality of life, SF-36 scores^a^: mean (s.d.)

	**Grampian**			**Wales**		
**Time**	**Nurse counsellor**	**Geneticist**	**Difference (95% CI)^b^**	**Nurse counsellor**	**Geneticist**	**Difference (95% CI)^b^**
*Physical functioning*						
Baseline	88.9 (18.6)	85.4 (21.8)		83.5 (21.8)	88.9 (18.3)	
FU1	88.2 (19.1)	88.6 (18.7)	−0.5 (−3.3 to 2.2)	84.9 (20.9)	88.0 (22.5)	0.3 (−3.7 to 4.3)
FU2	87.9 (18.5)	86.4 (21.3)	0.2 (−2.3 to 2.7)	84.6 (21.3)	88.6 (20.1)	−0.5 (−5.5 to 4.5)
						
*Social functioning*						
Baseline	84.0 (23.4)	84.6 (22.1)		77.7 (24.4)	79.8 (26.2)	
FU1	83.4 (23.9)	85.2 (21.2)	−0.6 (−5.7 to 4.5)	78.9 (24.8)	79.9 (25.4)	0.3 (−4.9 to 5.4)
FU2	84.7 (21.8)	87.2 (23.2)	−1.0 (−6.4 to 4.4)	78.0 (26.9)	80.1 (26.4)	−1.0 (−7.6 to 5.5)
						
*Role physical*						
Baseline	87.1 (29.0)	86.1 (31.3)		81.6 (33.5)	84.9 (30.9)	
FU1	86.8 (30.2)	85.1 (32.6)	5.1 (−1.6 to 11.8)^c^	77.4 (37.7)	82.7 (33.4)	−3.9 (−11.4 to 3.6)
FU2	86.0 (30.9)	87.1 (30.3)	1.3 (−6.1 to 8.7)	77.5 (37.9)	74.0 (38.7)	5.5 (−4.3 to 15.4)
						
*Vitality*						
Baseline	58.6 (21.3)	58.5 (23.3)		53.6 (21.1)	54.9 (21.3)	
FU1	60.8 (21.7)	61.7 (19.4)	0.5 (−3.7 to 4.7)	57.1 (22.3)	55.7 (20.3)	2.0 (−2.3 to 6.3)
FU2	61.6 (20.7)	63.9 (19.0)	−1.4 (−5.7 to 2.9)	55.3 (22.5)	58.3 (21.1)	−1.8 (−7.0 to 3.4)
						
*Bodily pain*						
Baseline	76.3 (23.9)	76.6 (25.1)		72.3 (25.4)	75.5 (25.0)	
FU1	78.6 (24.7)	77.4 (24.4)	2.3 (−2.4 to 7.1)	75.8 (24.6)	75.8 (26.0)	−0.2 (−5.3 to 4.9)
FU2	78.2 (24.5)	76.1 (23.8)	1.7 (−3.8 to 7.2)	74.9 (24.9)	75.2 (19.4)	−0.2 (−6.8 to 6.4)
						
*General health*						
Baseline	73.5 (19.8)	73.4 (18.9)		66.0 (20.6)	71.2 (20.0)	
FU1	75.2 (20.7)	74.9 (18.4)	0.8 (−2.5 to 4.0)	67.9 (21.4)	69.9 (20.7)	1.0 (−2.4 to 4.3)
FU2	75.0 (18.6)	73.7 (18.5)	1.7 (−2.0 to 5.4)	68.6 (21.5)	72.5 (19.4)	−0.03 (−3.9 to 3.8)

a Higher score indicates better health state (range 0–100).

b Adjusted for baseline.

c Larger than expected difference due to baseline imbalance of responders at FU1.

**Table 4:** Respondents indicating ‘Strongly Agree/ Agree’ with statements on causes of breast cancer, *n* (%)

	**Grampian**			**Wales**		
	**Nurse counsellor**	**Geneticist**	***P*-value^a^**	**Nurse counsellor**	**Geneticist**	***P*-value^a^**
*Stress is a major cause of breast cancer*						
Baseline	68 (36)	40 (42)		68 (35)	34 (35)	
Immediately after episode	80 (47)	39 (48)	0.98	63 (38)	31 (37)	0.98
Six months after episode	71 (42)	31 (44)	0.83	55 (37)	26 (36)	0.91
						
*One close relative with breast cancer always increases your risk considerably*						
Baseline	157 (83)	77 (81)		165 (84)	80 (83)	
Immediately after episode	127 (73)	52 (63)	0.12	130 (77)	65 (77)	0.99
Six months after episode	123 (77)	56 (76)	0.97	122 (81)	58 (80)	0.88
						
*Minor injury can cause breast cancer*						
Baseline	37 (20)	20 (21)		62 (32)	23 (24)	
Immediately after episode	38 (22)	22 (27)	0.50	50 (30)	16 (19)	0.09
Six months after episode	47 (29)	17 (23)	0.39	50 (33)	18 (25)	0.24

a From *χ*^2^ test (Yates' corrected – for 2 × 2 table).

**Table 5:** Comparison of health service costs per counselling episode

	**Grampian**			**Wales**		
**Group**	**Mean number of randomised appointments (range)**	**Unit cost^a^**	**Mean total cost per patient (£)**	**Mean number of randomised appointments (range)**	**Unit cost^a^**	**Mean total cost per patient (£)**
Nurse counsellor	1.26 (1–4)	108.01	136.55	1 (1–1)	140.10	140.10
Geneticist	1.18 (1–3)	125.99	148.30	1 (1–1)	127.60	127.60
Difference in cost (nurse counsellor–geneticist)			−11.54^b^			+12.50

a Unit cost of counselling appointment.

b (95% CI, £−25.43, £1.94).
